# COVID-19: Shining the Light on Africa

**DOI:** 10.4269/ajtmh.20-0380

**Published:** 2020-05-05

**Authors:** Philip J. Rosenthal, Joel G. Breman, Abdoulaye A. Djimde, Chandy C. John, Moses R. Kamya, Rose G. F. Leke, Matshidiso R. Moeti, John Nkengasong, Daniel G. Bausch

**Affiliations:** 1 Editor-in-Chief, American Journal of Tropical Medicine and Hygiene and Department of Medicine, University of California, San Francisco, San Francisco, California;; 2 President, American Society of Tropical Medicine and Hygiene and Fogarty International Center, National Institutes of Health, Bethesda, Maryland;; 3 Board Member, American Society of Tropical Medicine and Hygiene and Malaria Research and Training Center, Faculty of Pharmacy, University of Science, Techniques and Technologies of Bamako, Bamako, Mali;; 4 Past President, American Society of Tropical Medicine and Hygiene and Department of Pediatrics, Ryan White Center for Pediatric Infectious Diseases and Global Health, Indiana University School of Medicine, Indianapolis, Indiana;; 5 Department of Medicine, Makerere University, Kampala, Uganda;; 6 Infectious Diseases Research Collaboration, Kampala, Uganda;; 7 The Biotechnology Center, University of Yaoundé 1, Yaoundé, Cameroon;; 8 WHO Regional Director for Africa, Brazzaville, Congo;; 9 Africa Centres for Disease Control and Prevention (Africa CDC), African Union Commission, Addis Ababa, Ethiopia;; 10 Scientific Program Chair, American Society of Tropical Medicine and Hygiene and UK Public Health Rapid Support Team, Public Health England/London School of Hygiene and Tropical Medicine, London, United Kingdom

Africa has faced epidemics throughout its history, from smallpox to recurrent epidemics of malaria, sleeping sickness, and many other lethal diseases. The spread of HIV infection across the continent, beginning in the 1980s, caused tens of millions of deaths, and multiple localized outbreaks of Ebola virus disease, most notably in West Africa in 2013–2016, caused thousands of deaths and great suffering. Responses to African epidemics have been challenged by limited infrastructure and fragile healthcare systems, including the lack of adequate surveillance to assess the scope of the outbreak, and inadequate systems for the prevention, diagnosis, and management of disease. Yet Africa has also been the site of key advances in infectious disease control, including case containment and ring vaccination for smallpox; vector control and mass drug administration for onchocerciasis, malaria, and other vector-borne diseases; integrated community case management for common illnesses; effective treatment to facilitate prevention of HIV/AIDS and tuberculosis; and, most recently, development of treatments and a vaccine against Ebola virus disease. All countries, within Africa and beyond, have benefited from these advances.

The novel coronavirus disease (COVID-19) pandemic emerged in China in late 2019 and spread rapidly around the world in early 2020. The first confirmed case of COVID-19 in Africa was reported in Egypt on February 14 and the second a day later in Algeria. By March, COVID-19 cases were being reported across most of the continent. As with the rest of the world, initial cases were imported from other regions, followed by local transmission. By early April, nearly every country in Africa reported COVID-19, with hundreds to thousands of cases reported in the hardest-hit countries, presumably many additional infections that were unidentified, and hundreds of deaths from COVID-19 noted across the continent. While at this writing the number of cases and deaths in Africa remains relatively low, compared with other regions, numbers are increasing steadily, and fears are mounting of a major humanitarian crisis as transmission accelerates.

Africa has reacted quickly to COVID-19. An emergency meeting of African health ministers to discuss the pandemic was hosted by the African Union Commission on February 22, resulting in the rapid establishment of the Africa Taskforce for Coronavirus Preparedness and Response by the African Union Commission, Africa CDC, and the WHO Regional Office for Africa (AFRO), in partnership with African governments and other stakeholders. Africa Taskforce for Coronavirus Preparedness and Response identified and has worked diligently to deliver on six work streams: laboratory diagnosis and subtyping; surveillance, including screening at points of entry and cross-border activities; infection prevention and control in healthcare facilities; clinical management of severe COVID-19; risk communication; and supply chain management and stockpiles. The WHO has supported African countries in the development of public health response plans and, with the World Bank and other donors, established a global platform to link plans to funding, facilitating transparency with regard to financing of countries’ actions.

By March, governments and nongovernmental organizations announced programs to support global COVID-19 control and research. The Global Fund to Fight AIDS, Tuberculosis, and Malaria allowed recipients to use funds from existing grants for efforts to control COVID-19. No-interest loans for affected low-income countries were approved by the International Monetary Fund. Resources to fight COVID-19 were freed from funds for control of Ebola virus disease after abatement of an outbreak in the eastern Democratic Republic of Congo. On March 27, the African Development Bank launched a $3 billion social bond to address the pandemic, the largest bond ever issued by the bank. On April 7, the African Union and Africa CDC launched the Africa COVID-19 Response Fund, a public–private partnership with the AfroChampions Initiative, to raise funds for transmission prevention, medical responses, and socioeconomic support for vulnerable populations.

As has been the case elsewhere, national responses to the COVID-19 pandemic in Africa have varied. Initial responses, when the reported number of cases was low, were often muted. Over time, aggressive responses have been announced in many countries. In South Africa, an early and comprehensive program for widespread testing appears to be “flattening the curve” and may serve as a model for successful COVID-19 control. Nevertheless, formidable challenges remain in implementing response measures that appear to have allowed some resource-rich countries to gain the upper hand on COVID-19. What can Africa and global partners do to confront COVID-19?

1. *Community ownership and local action*. As with all outbreaks, large or small, community ownership and local action are imperative for success and must be at the heart of any response. Proper community engagement and involvement of social scientists will be essential in implementing local and universal solutions for urban and rural areas of Africa.
2. *Physical distancing*. Although it is essential to maintain physical distancing, novel solutions are needed for many areas of Africa, taking note of social and cultural norms. Stringent lockdowns and working from home may limit viral transmission, as appears to have been the case in some countries. However, lockdowns are unlikely to be implementable in many African countries, where a significant proportion of the population depends on performing physical labor for daily pay in a cash economy and where indoor plumbing, electricity, and Internet access are not always available. In these circumstances, sheltering at home may not be possible and may even enhance transmission in often overcrowded households and in urban slums. Informal urban settlements, refugee camps, and camps for internally displaced persons present additional challenges. Furthermore, in some areas with relatively little transmission of SARS-CoV-2, enforcement of lockdowns may entail greater risk to the population than the virus itself. There have been reports of violent enforcement of lockdowns, curfews, and travel bans, including deaths at the hands of police—vivid reminders that aggressive physical distancing can have unintended negative consequences. Food insecurity may become a major concern if the pandemic is protracted. A Malawi high court and political leaders in some other countries have suspended lockdowns because of lack of adequate provision for the poor. Africa, like all regions of the world, must look for solutions that best protect against viral transmission but respect civil liberties and the rule of law.
3. *Enhanced hygiene*. There is urgent need for aggressive implementation of low-cost, rapidly implementable sanitary measures, including improved access to soap, water, and hand sanitizer. Lessons can be learned from the control of Ebola virus disease, in which sanitation stations were established at the entry of virtually every office, shop, and place of worship.
4. *Laboratory testing*. The diagnostic market needs to be unlocked to ensure speedy and affordable access to diagnostics for Africa. African countries must have the infrastructure, including trained personnel, reagents, and equipment, to enable widespread COVID-19 testing, including molecular tests for acute diagnosis and serosurveys for surveillance to define infection patterns and guide prevention efforts. Africa CDC and the WHO have played an important role in providing testing equipment, kits, and laboratory training. The African Union plans to distribute one million COVID-19 test kits across the continent, but, as in many areas of the world, a severe shortfall remains.
5. *Supply chain*. The global supply chain is vital to provide critical medical and laboratory supplies, including personal protective equipment for healthcare workers, but is currently heavily fractured. This is especially problematic for Africa, considering the continent’s limited manufacturing capacity. There is need for reliable humanitarian aid corridors and services to assure provision of essential supplies until routine supply chains can be renewed. One short-term solution may be harvesting Africa’s vast capacity for local production of low-tech items, for example, production of reusable face masks by local seamsters.
6. *Enhanced patient care*. With limited care services, prevention must be the highest priority, but for those who fall ill, African countries must provide the best possible care, including isolation to prevent disease spread, and respiratory support for those critically ill. Shortages of intensive care unit beds and ventilators have been much discussed in the United States and Europe, but this conversation takes on a different tone for most of Africa. The WHO estimates that, for 43 of the continent’s 55 countries, there are fewer than 5,000 available intensive care unit beds, equating to about five beds per one million people (compared with 4,000 beds per million in Europe). At least 10 countries have no ventilators. Furthermore, in most countries, there is a critical shortage of healthcare workers trained in intensive care. Novel solutions to enable ventilation of ill patients in limited resource environments are desperately needed, possibly building on recent efforts in pandemic-stretched high-income countries. Expanded use of supplemental oxygen, continuous positive airway pressure, and prone positioning may minimize the need for mechanical ventilation, although it is important to maintain vigilance in avoiding transmission to healthcare workers with these procedures.
7. *Risk communication*. Effective communication advocating measures such as physical distancing, masks, and hygiene is essential, and should take advantage of the social connectedness of many African societies. Affordable local radio and television stations, traditional village announcers, religious leaders, community relays, and social media should be used for widespread public health messaging, and in particular to counter the expected spread of rumor and misinformation (which thrives in a communication vacuum) and lack of participation by segments of the population who do not perceive themselves to be at risk. Existing networks may be used, for example, established groups of people living with HIV.
8. *Modeling*. Mathematical models have been used around the world to guide COVID-19 preparation and response. Despite their limitations, these tools should be rapidly extended to support African countries in the same manner.
9. *Maintenance of services for non–COVID-19* *diseases*. Public health measures to control the spread of COVID-19 must engender minimal disruption of standard care for other infectious and noninfectious diseases. During the West African outbreak, disruptions and transfers of resources due to Ebola virus disease were estimated to have resulted in thousands of excess maternal and infant deaths and millions of untreated cases of malaria. A similar pattern is looming in the response to COVID-19. In late March 2020, because of the pandemic, UNICEF acknowledged that temporary discontinuation of mass vaccination campaigns might be necessary. Although this move may be prudent to limit viral transmission, it will undoubtedly lead to excess cases of vaccine-preventable diseases. UNICEF urged prompt resumption of immunization activities as soon as is possible. Consistent with UNICEF guidance, routine immunization services should be a high priority, even in the setting of lockdowns. Maintenance of maternal child health and surgical services is clearly at risk. Similarly at risk are management of chronic infections that require regular chemotherapy, notably HIV infection and tuberculosis, and acute infections that require rapid treatment, in particular malaria. Systems for the prevention and treatment of many other tropical infections, such as schistosomiasis, filarial infections, trypanosomiasis, and measles, are fragile, and may be seriously disrupted. Noncommunicable diseases are increasing across the continent, and maintaining surveillance for and management of hypertension, heart disease, diabetes, cancer, and other diseases will be challenged. Ironically, many chronic noninfectious diseases for which management is challenged by the pandemic are risk factors for poor outcomes in COVID-19. Considering that the risk of severe and fatal disease is highest in the elderly, the young population structure of most African countries may be an advantage. Whether this advantage will be counterbalanced by inadequately managed comorbidities remains to be seen; continued attention to controlling these comorbidities, including chronic infectious and noninfectious diseases, is a high priority.
10. *Therapeutics and vaccines.* As everywhere in the world, decisions on prevention and management of COVID-19 in Africa must be evidence based. For example, use of chloroquine, a common antimalarial that has been readily available in Africa for decades and which has been advocated as a treatment for COVID-19 by politicians and other nonexperts, should be limited to clinical trials until efficacy and safety are better characterized. Despite this recommendation, use of chloroquine and its analog hydroxychloroquine for COVID-19 treatment and prevention will likely occur, as in other parts of the world, with attendant risks of shortages of the drugs for appropriate indications (non-*falciparum* malaria and rheumatologic diseases) and of flooding markets with fake and substandard medicines. Ill-informed comments suggesting Africa as the location for COVID-19 vaccine testing have unfortunately stirred up resistance to all vaccines. Governments and policy-leaders on the continent should continue to adhere to WHO guiding principles for immunization during the pandemic.
11. *Equitable access*. It is essential that any therapeutics, vaccines, and diagnostic assays validated through rigorous scientific study are made available in Africa. In the long term, this will require increases in manufacturing capacity in Africa, but in the short term, it will depend on establishing ethical and legal frameworks of inclusion. Provisions for equitable access and pricing, facilitated by advances with other diseases such as HIV infection, will need to be addressed. Within African countries, equity requires attention to the most vulnerable members of society, in particular mothers and young children.
12. *Research*. Research on the epidemiology, pathogenesis, genetics, prevention, diagnosis, and management of COVID-19 in the African context will be important to provide guidance for ongoing approaches to the pandemic. The leadership of African CDC, the WHO/AFRO, the African Academy of Sciences, the National Academies of Sciences, African Ministries of Health and Ministries of Scientific Research, and other partners in developing research priorities and protocols will be critical to advancing research on COVID-19 to best guide future policies. Adapting and applying the WHO Research and Development Blueprint for COVID-19 to the African context is a logical place to start, with the potential corollary benefit of long-term enhancement of research infrastructure on the continent.
13. *Addressing the economic impact*. Beyond medical consequences, COVID-19 will have a profound economic impact on Africa, a continent much less prepared to absorb economic downturns than wealthier regions that have to date primarily borne the brunt of the pandemic. The African Development Bank recently estimated that COVID-19 may cost Africa tens of billions of dollars in gross domestic product, increases in national budget deficits of 3–5%, and overall increases in public debt of more than $100 billion. The African Union estimates the loss of 20 million jobs on the continent. Over the course of the pandemic, African countries already suffering from resource limitations must address the same economic challenges engaging the entire world, with loss of income most threatening to the most vulnerable members of society. We must avoid the initiation of a vicious cycle, with viral infections feeding economic and societal disruption, which feed additional viral infections.

Novel coronavirus disease presents unique and grave challenges for Africa, as it has for the rest of the world. Infrastructure and resource constraints make COVID-19 an even more threatening disease for Africa than for many of the resource-rich countries that it has already devastated. However, multiple African countries and organizations have demonstrated leadership in elaborating systems for the prevention of COVID-19 and in planning for optimal research strategies in the African context. Key public health, clinical, and research goals to mitigate the impact of COVID-19 can be achieved. Ideally, progress toward the mitigation of COVID-19 will lead to long-term improvements in African health and research systems. But, in the short term, much additional support from international and African sources is needed to prevent COVID-19 from becoming an increasing health and economic disaster in Africa.

